# Effectiveness of interventions by non-professional community-level workers or family caregivers to improve outcomes for physical impairments or disabilities in low resource settings: systematic review of task-sharing strategies

**DOI:** 10.1186/s12960-023-00831-7

**Published:** 2023-06-21

**Authors:** Anne Kumurenzi, Julie Richardson, Lehana Thabane, Jeanne Kagwiza, Gerard Urimubenshi, Leah Hamilton, Jackie Bosch, Tiago Jesus

**Affiliations:** 1 School of Rehabilitation Sciences, McMaster University, Hamilton, Canada; 2 College of Medicine and Health Sciences, University of Rwanda, Kigali, Rwanda; 3 Health Research Methods, Evidence and Impact, McMaster University, Hamilton, Canada; 4 Biostatistics Unit, St Joseph’s Healthcare Hamilton, Hamilton, Canada; 5 Faculty of Health Science, University of Johannesburg, Johannesburg, South Africa; 6 Population Health Research Institute, Hamilton, Canada; 7 Occupational Therapy, School of Rehabilitation Science, McMaster University, Hamilton, Canada; 8 Feinberg School of Medicine, Northwestern University, Evanston, United States of America

**Keywords:** Systematic review, Physical disabilities, Community health workers, Non-healthcare providers, Health volunteers, Family caregivers, Physical function, Adults, Low-resource settings

## Abstract

**Background:**

In low-resource settings, access to basic rehabilitation could be supplemented by community-level interventions provided by community health workers, health volunteers, or family caregivers. Yet, it is unclear whether basic physical rehabilitation interventions delivered to adults by non-professional alternative resources in the community, under task-shifting or task-sharing approaches, are effective as those delivered by skilled rehabilitation professionals. We aim to synthesize evidence on the effectiveness of community-level rehabilitation interventions delivered by non-professional community-level workers or informal caregivers to improve health outcomes for persons with physical impairments or disabilities.

**Methods:**

We performed a systematic review with a PROSPERO registration. Eight databases were searched for (PubMed, CINAHL, Global Health, PDQ Evidence, Scopus, ProQuest, CENTRAL, and Web of Science), supplemented by snowballing and key-informant recommendations, with no time restrictions, applied. Controlled and non-controlled experiments were included if reporting the effects of interventions on mobility, activities of daily living (ADLs), quality of life, or social participation outcomes. Two independent investigators performed the eligibility decisions, data extraction, risk of bias, and assessed the quality of the evidence using the GRADE approach.

**Results:**

Ten studies (five randomized controlled trials [RCTs]) involving 2149 participants were included. Most common targeted stroke survivors (*n* = 8); family caregivers were most frequently used to deliver the intervention (*n* = 4); and the intervention was usually provided in homes (*n* = 7), with training initiated in the hospital (*n* = 4). Of the four RCTs delivered by family caregivers, one demonstrated a statistically significant improvement in mobility (effect size: 0.3; confidence interval [CI] 121.81–122.19; [*p* = 0.04]) and another one in ADLs (effect size: 0.4; CI 25.92–35.08; [*p* = 0.03]). Of the five non-RCT studies by community health workers or volunteers, one demonstrated a statistically significant improvement in mobility (effect size: 0.3; CI 10.143–16.857; [*p* < 0.05]), while two demonstrated improved statistically significant improvement in ADLs (effect size: 0.2; CI 180.202–184.789 [*p* = 0.001]; 0.4; CI − 7.643–18.643; [p = 0.026]). However, the quality of evidence, based on GRADE criteria, was rated as low to very low.

**Conclusions:**

While task-sharing is a possible strategy to meet basic rehabilitation needs in low-resource settings, the current evidence on the effectiveness of delivering rehabilitation interventions by non-professional community-level workers and informal caregivers is inconclusive. We can use the data and experiences from existing studies to better design studies and improve the implementation of interventions.

*Trial registration* PROSPERO registration number: CRD42022319130

**Supplementary Information:**

The online version contains supplementary material available at 10.1186/s12960-023-00831-7.

## Background

Physical rehabilitation interventions can optimize function and minimize disability for those with physical impairments [1] but are often inaccessible to populations living in low-resource settings [2–4]. A growing burden of health conditions that lead to physical impairments has been observed in low-resource countries [5], wherein the Years Lived with Disability amenable to physical rehabilitation interventions more than doubled from 1990 to 2017 [6]. However, rehabilitation service provision and skilled human resources remain scant in low-resource settings [5, 7]. Here, we follow the standpoint that low-resource settings are not limited to low or middle income countries (LMICs) but include settings with structural health resource limitations, including financial shortages (of the system or those accessing the system), suboptimal service delivery systems, undeveloped physical infrastructure, or human resources limitations in workforce size or skills [8].

In high-resource settings, physical rehabilitation is usually provided by credentialed, skilled health professionals, such as (but not limited to) rehabilitation physicians, rehabilitation psychologists, physiotherapists, occupational therapists, speech and language therapists, orthotists and prosthetists, and nurses. However, in low-resource settings, the availability of skilled rehabilitation workers is insufficient to meet the high and increasing population needs [7]. In low-resource settings, non-professional community-level health workers or informal caregivers may provide a valid and feasible alternative, extension, or complement to the care provided by rehabilitation specialists. These non-professional human resources include community health workers (CHWs), Accredited Social Health Activists in India, family caregivers, health volunteers, and lay personnel [6, 9–11]. These alternative resources are essential for the deployment of “task-shifting” and “task-sharing” approaches likely needed to improve population access to basic rehabilitation in low-resource settings [12, 13]. In these approaches, skilled health care workers train, provide support or oversight to the non-professional community-level workers or informal caregivers [14, 15]. Yet, it is unclear whether basic rehabilitation interventions delivered by non-professional human resources are effective.

Currently, rehabilitation in low-resource community settings is mainly provided through non-governmental organizations or community-based rehabilitation (CBR) approaches, often a part of the formal health sector. CBR is a cross-sectoral, community-level approach to addressing the health but also the educational, social, and other holistic needs of people with disabilities [16]. Two systematic reviews addressed the effectiveness of CBR in low-resource contexts [16, 17]; however, these reviews include interventions and outcomes that are not necessarily health-oriented (e.g., focused on social inclusion and economic dimensions) [16, 17], did not focus exclusively on the effectiveness of health interventions (e.g., including qualitative studies [17]), included a wide range of people with disabilities, such as those arising from mental or intellectual impairments [16], and finally did not include recent studies (published in 2012 and 2016) [16, 17]. Our focus is specifically on the effectiveness of health-based interventions for the rehabilitation of physical impairments or disabilities, excluding those arising from mental health and intellectual conditions—as the scope of the health interventions, health outcomes, and the skill set of the health workforce vary.

Our primary study question is:

- Are physical rehabilitation interventions delivered by non-professional community-level workers or informal caregivers effective in improving physical functioning (mobility, activities of daily living [ADLs])?

Our secondary research questions are:

- What are the characteristics of the interventions that demonstrated an effect?
- Are the physical rehabilitation interventions delivered by non-professional community-level workers or informal caregivers effective in improving other health-related or health system outcomes, such as quality of life (QOL), social participation, self-management behaviors, service access and service utilization, and in improving key care processes (e.g., care coordination for community transitions).

## Methods

The systematic review protocol was registered with PROSPERO (CRD42022319130). The reporting of this review follows the Preferred Reporting Items for Systematic Reviews and Meta-Analyses (PRISMA) checklist [18]—see Additional file 1: Appendix S1. In addition, the GRADE approach [19] was used to assess the quality evidence of studies.

### Search strategy

Eight databases were searched: PubMed, CINAHL (through EBSCO), Global health (through EBSCO), PDQ Evidence, Scopus, ProQuest, the Cochrane Central Register of Controlled Trials (CENTRAL), and Web of Science. No time restrictions were applied. Additional file 1: Appendix S2 provides a complete search strategy for each of the eight databases. In short, the search strategy combined alternative sets of keywords and indexed terms for: (1) non-professional community-level workers or informal caregivers (CHWs, health volunteers, family caregivers, lay personnel) or community-level forms of service delivery; (2) rehabilitation service, physical function, disability, or related outcomes; (3) low-resource settings in any country as well as entire LMICs; (4) study types addressing the efficacy or effectiveness of programs or interventions; (5) adult populations; and (6) the exclusion of articles focused on mental health conditions or psychiatric rehabilitation. In addition, reference lists from included studies and published systematic reviews on partly related topics (e.g., CBR) were screened for references (snowballing). Finally, supplied with our preliminary list of the inclusions, three key informants (e.g., external scholars) who had published on community-level or CBR topics, respectively, in Africa, Asia, and Latin America were also contacted to determine if there were any unpublished or undetected studies relevant to the review.

### Eligibility criteria

#### Population

We included studies of adults (aged 18 and older) with physical impairments or disabilities from possibly debilitating health conditions such as chronic non-communicable diseases (e.g., stroke, cancer, respiratory conditions, arthritis, low back pain), traumatic injuries (e.g., head injuries, spinal cord injuries), or communicable diseases (e.g., HIV/AIDs) and that were conducted in low-resource settings as defined by Van Zyl et al. [8]. The option to address low-resource settings overall expands from our early registered protocol definitions focused on LMICs. We excluded studies of adults that focused on impairments or disabilities secondary to mental health or cognitive deterioration.

#### Interventions

We included studies of physical rehabilitation interventions delivered by non-professional community-level workers or volunteers (e.g., CHWs, community/health volunteers, lay workers), or informal caregivers in the community (e.g., community centers) or home-based settings, either individually or in groups, initiated, trained, or supervised by skilled health professionals (i.e., the “task-shifting” or “task-sharing” component).

#### Control/comparator(s)

Any comparator/control (such as usual or conventional care with follow-up), active or passive, was accepted. We also included non-controlled intervention studies (pre- and post-test).

#### Outcomes

Studies were included that reported on at least one of the following study outcomes: physical functioning (mobility, ADLs) as primary outcomes or QOL or social participation as secondary outcomes.

#### Study type

We included randomized controlled trials (RCTs), non-randomized controlled experiments, non-controlled experiments (e.g., pre- and post-test designs; interrupted time series), and longitudinal observational studies (cohort studies, case–control studies) on the impact of a program or intervention.

#### Language

No restrictions were applied to the language of the full texts, provided that a title and abstract were available in English, French, Spanish, or Portuguese. Collectively, the research team had the capacity to review papers in these languages.

#### Time

No time restrictions were applied to the date of study publication.

### Selection of studies

Titles and abstracts of studies detected by the searches were uploaded to a systematic review software: COVIDENCE (Melbourne, Australia) [20]. First two independent reviewers (AK and JB) screened titles-and-abstracts. Then, two independent reviewers (reviewer 1: AK; reviewer 2: LH or GU) performed the full-text assessments, followed by one round of reviewers’ discussion toward agreement; the senior authors (TJ and JB) decided on any prevailing disagreements.

### Data extraction

The following data were extracted: country/setting, study design, participants (sample size, number of groups in the intervention, health condition/disability, demographic characteristics [age, sex/gender]), intervention (type, personnel providing intervention, setting), outcomes measures, and study’s outcomes. Two independent reviewers (reviewer 1: AK; reviewer 2: LH or GU) performed data extraction as adapted from the Cochrane Consumers and Communication Review Group’s Data Extraction Template for Cochrane Reviews [21] and the COVIDENCE tool for data extraction.

### Risk of bias assessment

For RCTs, we used the Cochrane handbook for systematic reviews [22], and reviewer 1: AK; reviewer 2: LH or GU rated each study as either low, unclear, or high risk of bias for each domain and provided explanations to justify. For non-RCTs, we used the ROBINS-I tool [23], and reviewer 1: AK; reviewer 2: LH or GU rated each study as either low, moderate, serious, critical risk of bias and no information on which to base the judgment.

### Grading strength evidence

The quality of evidence and recommendations was further assessed and graded using the Grading of Recommendations Assessment, Development and Evaluation (GRADE) guidelines as “high”, “moderate”, “low” or “very low”. The quality of evidence assessments was performed by two independent reviewers (reviewer 1: AK; reviewer 2: LH or GU), with a consensus reached after discussions with the senior authors (TJ and JB).

### Synthesis

Due to the heterogeneity of the studies (in study design, intervention details, outcome measures), a meta-analysis was not possible. Therefore, we performed a tabular and narrative synthesis of the results, organizing findings by RCTs and non-RCTs.

## Results

**Fig. 1:**
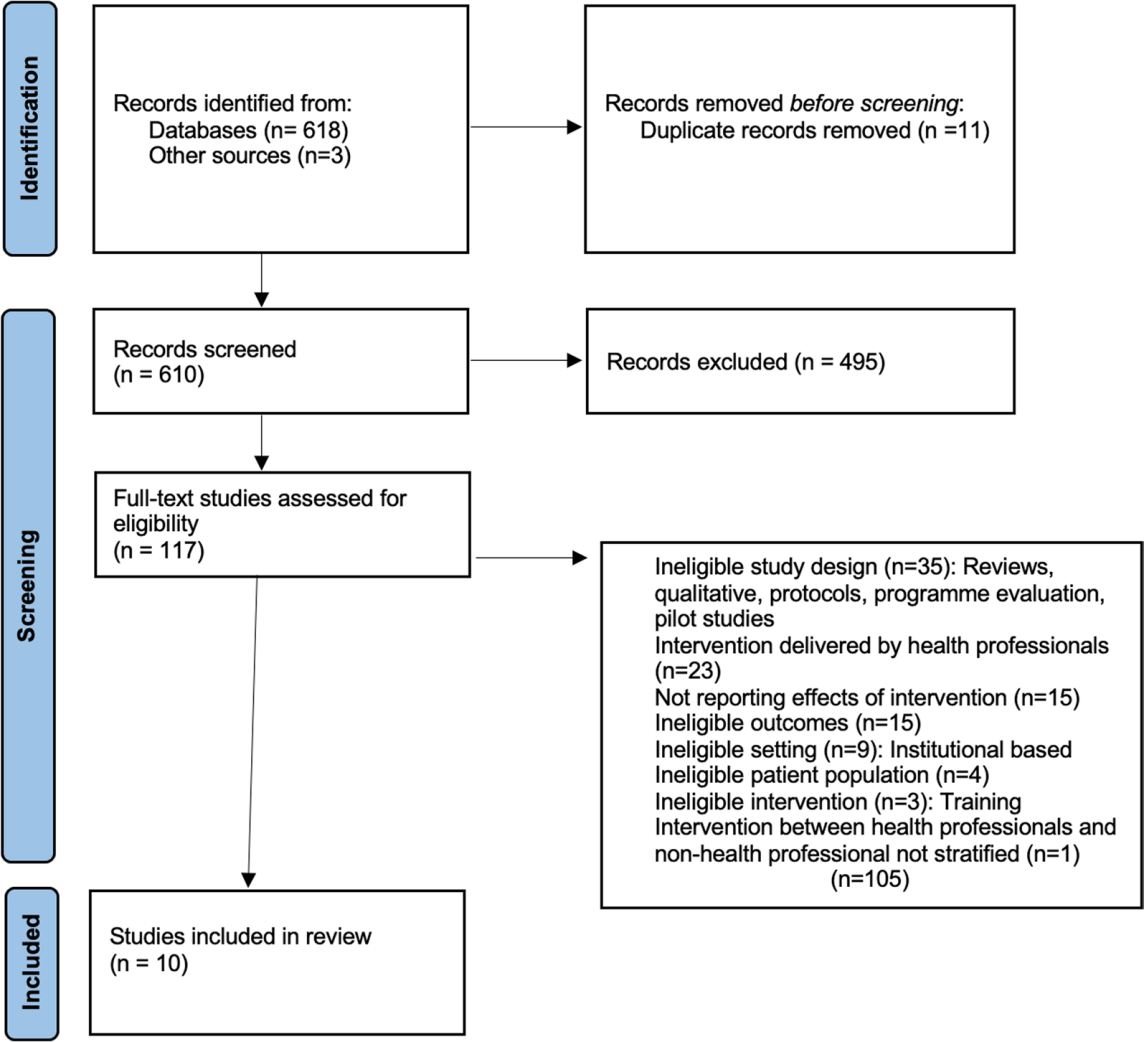
PRISMA flow diagram

Figure 1 provides the PRISMA flowchart of this review. From 610 deduplicated records, 117 underwent full-text screening; ten were eligible for inclusion. The most common reasons for exclusion were ineligible study designs, interventions delivered primarily by health professionals, and studies not reporting the effect of results.

### Study characteristics

Table 1 describes the Population, Intervention, Comparator and Outcome types (PICOs) as well as the country, study design, and the key findings of each of the ten included studies: five were RCTs [24–28] (Table 1a), and five were non-RCT studies: one non-randomized controlled experiment [29], three pre- and post-test designs [30–32], and one comparative observational study [33] (Table 1b). Studies were published between 2001 [28] and 2021 [30], with the majority of studies conducted either in Thailand (*n* = 3) or China (*n* = 3). Sample sizes varied from 11 [30] to 1250 [26], with 2149 participants included in this review. Stroke was the most frequently addressed condition (*n* = 8). Family members (*n* = 4) and village health volunteers (*n* = 3) were the personnel most frequently used to deliver the intervention. The most common setting where rehabilitation was provided was the patients’ home (*n* = 7), of these, four provided initial training of trainers in the hospital. All five RCTs compared interventions to usual care (passive), and most studies assessed mobility (*n* = 5), ADLs (*n* = 5), and QOL (*n* = 5).

**Table 1:** Summary of study characteristics and main findings

Author and year	Methods (country, design)	Participant: sample size, age (mean or mean and SD), female %, primary diagnosis (Dx)	Intervention (type, personnel, setting, brief description of intervention)	Comparison	Follow-up period	Outcomes and tools	Key findings at 6 months		
							Inter*	Cont*	*p* value
a: RCTs									
Interventions by family caregivers									
Chu et al., 2020	China, RCT	*N* = 61 Age: intervention 62.84 (9.71), control 66.17 (8.51) Female: intervention 68%, control 53% Dx: stroke, ischemic 59%	Family-caregiver delivered, in-homes after training in hospital Mobility: transfers, walking, stair climbing ADL: grooming, dressing, bathing Continence: bowel and bladder control, toileting	Conventional care, no post-discharge rehabilitation	6 months	ADLs: BI (0–100) Mean (SD)	72.6 (21.18)	61.00 (34.63)	0.03
						QOL: EQ-5D Mean (SD)	1.55 (0.39)	1.56 (0.54)	0.91
Zhou et al., 2019	China, RCT	*N* = 244 Age: intervention 64.3, control 66.2 Female: intervention 54%, control 56% Dx: stroke, ischemic 73%	Family-caregiver delivered, in-home after training in hospital Mobility: bed, balance, walking ADL: grooming, dressing, bathing Continence: bowel and bladder control, toileting	Conventional care (no post-discharge rehabilitation)	6 months	Walking: FAC (0–5) Mean (SD)	4.6 (1.7)	5.0 (1.4)	0.04*
						ADLs: BI (0–100) Mean (SD)	70.1 (25.5)	74.1 (23.0)	*p* = 0.27*
						QOL: EQ-5D (0–1) Mean (SD)	0.7 (0.3)	0.8 (0.3)	0.15*
Lindley et al., 2017	India, RCT	*N* = 1250 Age: intervention 57.5 (12.92), control 58 (14.21) Female: intervention 68%, control 66% Dx: stroke, ischemic 77%	Family-caregiver delivered, in-homes after training in hospital Mobility: limb positioning ADL: task specific activities	Post-discharge care (no therapy to some outpatient therapy sessions)	6 months	Mobility: EQ-5D-3L (proportion with some or severe deficits)	237/529 (45%)	228/510 (45%)	0.32
						ADLs: BI (0–100) Mean (SD)	82.1 (23.09)	82.6 (23.19)	0.74
						ADLs: NEADL (0–66) Mean (SD)	31.0 (17.67)	31.2 (17.52)	0.86
						Selfcare: EQ-5D-3L (proportion with some or severe deficits)	251/529 (47%)	230/510 (45%)	0.75
Ozdemiret al., 2001	Turkey, RCT	*N* = 60 Age: intervention 61.8 (9.2), control 59.1 (5.9) Female: control 30%, intervention 63% Dx: stroke, hemorrhagic 77%	Family-delivered, in-homes after training in hospital Mobility: convenient bed positioning and exercises. Splints, orthoses, and devices were also provided	Therapeutic exercises and neuromuscular facilitation exercises, physical agents	5 months	Function independence: FIM (18–126), mean change score (SD)	59.63 (14.19)	12.30 (13.38)	0.001
Interventions by community health workers									
Cobbing et al., 2017	South Africa, RCT	*N* = 76 Age: intervention 43.4, control 44.7 Female: intervention 76.3%, control 76.3% Dx: HIV	CHWs-delivered in-homes Mobility: aerobic exercises, strength and stretch (upper and lower limbs) and functional exercises (sitting to standing and bridging) and walking	Conventional care, no rehabilitation in the community	6 months	Mobility: WHODAS (0–4), mean (SD)	0.18 (0.56)	0.21 (0.59)	> 0.05
						Mobility: RMI (0–15) Mean (SD)	14.24 (1.50)	13.82 (2.33)	> 0.05
						Walking: 6MWT (distance), mean (SD)	327.71 (73.61)	303.29 (92.48)	> 0.05
						QOL: WHOQOL (1–5), mean (SD)	3.62 (0.70)	3.41 (0.93)	> 0.05

Author and year	Methods (country, design)	Participant: sample size, age (mean or mean and SD), female %, primary diagnosis (Dx)	Intervention (type, personnel, setting, brief description of intervention)	Comparison	Follow-up period	Outcomes and tools	Key findings		
							CRAT	Cont	*p*
b. Non-RCTs									
Interventions delivered by non-professional community health workers or volunteers									
Ru et al., 2017	China, non-randomized controlled experiment	*N* = 365 Age: 61.9 (9.5) Female: 28.9% Dx: stroke, cerebral hemorrhage: 70 (20%)	CRAT by community rehabilitation workers and family caregivers in community centers and patients’ home Mobility: lying, sitting, sitting-to-standing, and standing	No special intervention	3 months	ADLs: BI (0–100) (proportion of severity of disease: ≥ 3 and < 4; ≥ 4) Mean (SD)	82.3 (21.3)	76.7 (23.5)	0.001
							84.2 (21.9)	79.9 (21.7)	0.001
						Function: Fugl–Meyer motor function assessment (0–100) (proportion of severity of disease: ≥ 3 and < 4; ≥ 4) Mean (SD)	76.7 (23.5)	58.7 (28.9)	0.001
							66.9 (25.4)	57.6 (26.7)	0.023

Author and year	Methods (country, design)	Participant: sample size, age (mean or mean and SD), female %, primary diagnosis (Dx)	Intervention (type, personnel, setting, brief description of intervention)	Comparison	Follow-up period	Outcomes and tools	Key findings		
							Pre-test	Post-test	*p* value
Chinchai et al., 2021	Thailand, pre- and post-test	*N* = 11 Age (range): 41–80 Female: 27% Dx: stroke	Rehabilitation education by VHWs in community rehabilitation centers Fundamental knowledge of stroke Mobility: physical exercise and gait training, UE function training ADL: selfcare, transfers, home chores	No control group	2 months	Basic ADLs: the ADL assessment tool (BADL) (23–155) Mean (SD)	67.41 (23.31)	75.50 (21.17)	0.026
Chinchai et al., 2020	Thailand, pre- and post-test	*N* = 25 Age (range): 30–80 Female: 40% Dx: stroke	Rehabilitation education by VHWs in community rehabilitation centers Basic knowledge of stroke, mobility: exercise and gait, transfers, UE functional training ADL: dressing, grooming	No control group	3 months	QOL: WHOQOL–BREF–THAI (0–100) Mean (SD)	71.44(8.38)	84.88(12.07)	0.000
						Community integration: CIQ (0–29) Mean [SD])	9.80 (3.96)	11.44 (4.68)	0.006
Chinchai et al., 2017	Thailand, pre and post-test	*N* = 27 Age range: 30–80 Female: 37% Dx: stroke	Rehabilitation education by VHWs in patients’ homes Basic knowledge of stroke, mobility: gait training, balance, and UE and LE functional training	No control group	2 months	Walking: 10-m walk test Mean (SD)	34.73 (8.48) 0.17 m/s	32.18 (9.32) 0.19 m/s	*p* < 0.05
						UE function: FMA (0–44) Mean (SD)	36.81 (9.59)	37.26 (9.67)	0.474
Balasubramanian et al., 2012	India, comparative observational	*N* = 30 Age: IBR 37 (18.13), CBR 54 (13.55) Dx: locomotor disabilities	IBR by healthcare professionals*	CBR by CBR workers	Not described^a^	Function: FIM (18–126) Mean (SD)	IBR: 117.40 (3.04)	CBR: 111.60 (12.02)	> 0.05
						QOL: WHOQOL–BREF: (1–5) Mean (SD	IBR: 4.20 (0.414)	CBR: 4.00 (1.604)	> 0.05

*SD* standard deviation, *RCT* randomized control trial, *Inter.* intervention, *Cont.* control, *ADLs* activities of daily living, *QOL* Quality of life, *NEADL* Nottingham extended ADL scale, *FAC* functional ambulation category, *HIV* human immunodeficiency virus, *CHWs* community health workers, *WHODAS* world health organization disability assessment schedule, *RMI* Rivermead mobility index, *6MWT* 6 min walk test, *WHOQOL* world health organization quality of life, *PT* physiotherapy, *OT* occupational therapy, *FIM* functional independence measure, *CRAT* community-based rehabilitation appropriate technique, *UE* upper extremity, *LE* lower extremity, *VHVs* village health volunteers, *WHOQOL–BREF* World health organization quality of life–BREF, *CIQ* community integration questionnaire *FMA* Fugl–Meyer assessment, *IBR* institutional-based rehabilitation, *CBR* community-based rehabilitation

*p value adjusted for confounders

^a^Description of intervention not described

### Quality appraisals

**Fig. 2:**
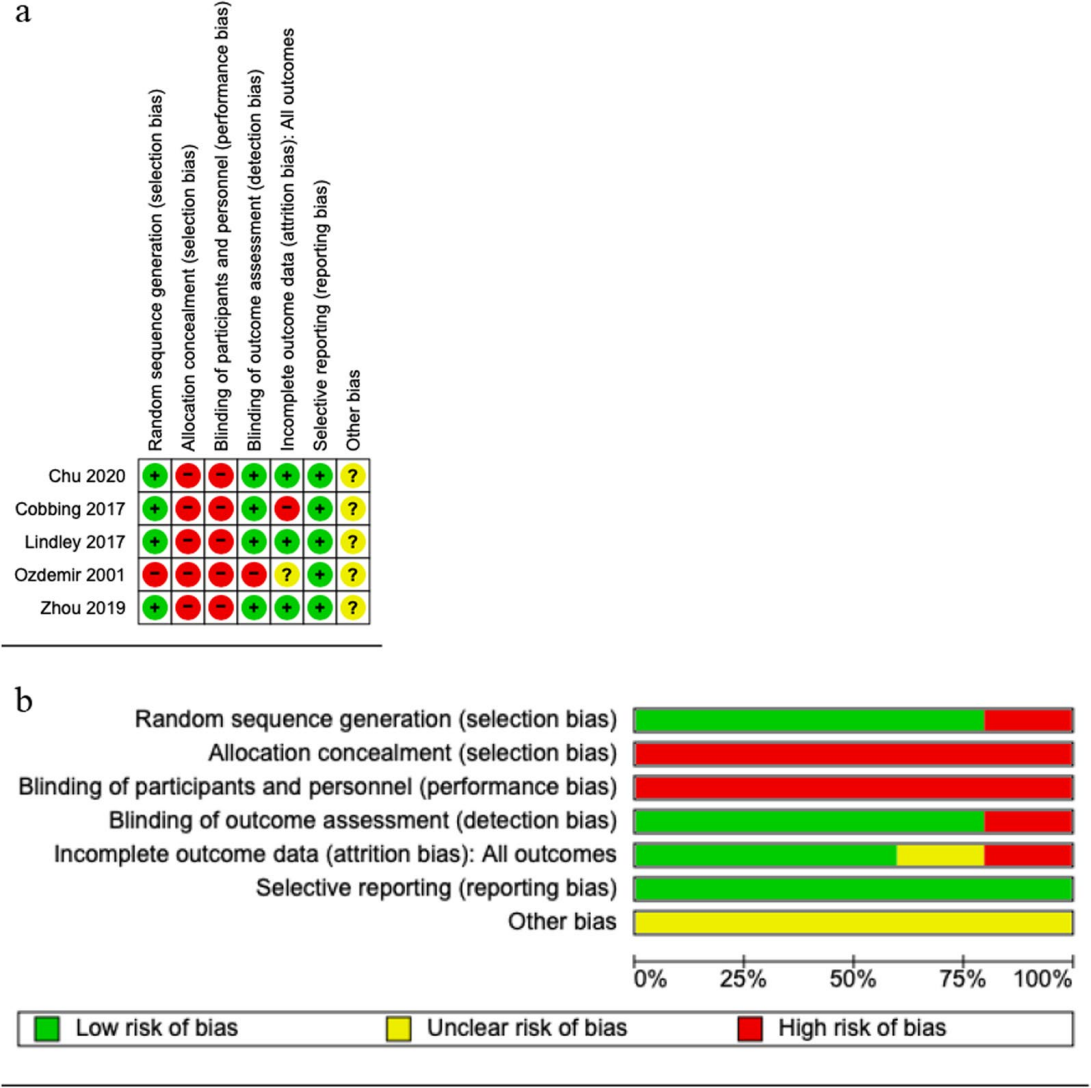
Cochrane risk of bias assessment. **a** For individual RCTs. **b** Overall. Red (−): high risk of bias; Yellow (?): unknown risk of bias; Green (+): low risk of bias

For the RCTs, Fig. 2a shows the risk of bias within RCTs, while Fig. 2b shows the risk of bias across the RCTs; detailed justifications for individual RCT assessments are presented in Additional file 1: Appendix S3. In a synthesis, none of the five RCTs had information on the concealment of allocations prior to assignment. In turn, one did not blind outcomes assessors [28]. While none of the RCTs blinded participants and personnel, that is inherent to most studies of rehabilitation interventions.

For the non-RCTs, Table 2 shows their appraised risk of bias detailed justifications for individual study assessments are presented in Additional file 1: Appendix S4. The only non-randomized controlled experiment [29] had mixed risk of bias appraisals (e.g., from a *low* risk of bias due to confounding to a *serious* risk of bias in measuring outcomes). The other four studies, i.e., three pre- and post-test designs and one comparative observational study [30–33], were appraised as having a *serious* or *critical* risk of bias (or *no information* to determine the risk) in all assessed domains; the single exception was a *low* risk of bias in one criterion (i.e., the selection of the reported results) of one particular study [30].

**Table 2:** Risk of bias for non-RCTs

Non RCTs (Cochrane risk of bias [ROBINS-I])							
Study	Pre-intervention domains		At intervention domain	Post-intervention domains			
	Bias due to confounding	Bias due to selection of participants	Bias due to classification of interventions	Bias due to deviations from intended interventions	Bias due to missing data	Bias in measurement of outcomes	Bias in selection of the reported results
Ru et al. 2017	Low risk	Serious risk	Moderate risk	No information	Moderate risk	Serious risk	Low risk
Chinchai et al. 2021	Serious risk	Critical risk	No information	Critical risk	Serious risk	Serious risk	Low risk
Chinchai et al. 2020	Serious risk	Critical risk	No information	No information	Serious risk	Serious risk	Serious risk
Chinchai et al. 2017	Serious risk	Critical risk	No information	No information	Serious risk	Serious risk	Serious risk
Balasubramanian et al. 2012	Serious risk	Critical risk	Critical risk	No information	No information	Serious risk	Serious risk

Low risk of bias: the study is comparable to a well-performed randomised trial with regard to this domain

Moderate risk of bias: the study is sound for a non-randomised study with ﻿regard to this domain but cannot be considered comparable to a well-performed randomised trial

Serious risk of bias: the study has some important problems

Critical risk of bias: the study is too problematic in this domain to provide any useful evidence on the effects of intervention

No information: on which to base a judgement about risk of bias for this domain

Finally, based on the GRADE criteria, Table 3 presents an outcomes-based summary of findings stratified by RCTs and non-RCTs. For the mobility, ADLs, and QOL outcomes in randomized trials, the confidence in the strength of the evidence on the effectiveness of the tested interventions was all appraised at a *low* quality. In contrast, the observational studies were appraised at a *very low* quality.

**Table 3:** Quality of the evidence included in the review (GRADE)

Certainty assessment						№ of patients		Quality of the evidence (GRADE)	Comment
№ of studies	Study design	Risk of bias	Inconsistency	Indirectness	Imprecision	Intervention	Usual care		
Randomized trials									
Mobility (assessed with FAC, EQ-5D, WHODAS; follow-up: 6 months)									
3	Randomised trials	Not serious^a^	Serious^b^	Not serious^c^	Serious^b^	779	793	⨁⨁◯◯ Low	One study reported an effect after adjusting for confounders (Zhou et al.), effect size of 0.3, CI 121.81–122.19; (*p* = 0.04)
Activities of daily living (assessed with BI; follow-up: 6 months)									
3	Randomised trials	Not serious^a^	Serious^d^	Not serious^e^	Serious^d^	772	785	⨁⨁◯◯ Low	One study reported an effect (Chu et al.) effect size of 0.40, CI 25.92–35.08; (*p* = 0.03)
Quality of life (assessed with EQ-5Q and WHOQOL; follow-up: 6 months)									
3	Randomised trials	Not serious^a^	Serious^f^	Not serious^c^	Serious^f^	187	196	⨁◯◯◯ Very low	All studies showed no effect of intervention
Non-RCT studies									
Activities of daily living (assessed with BI and BADL assessment tool; follow-up: 2–3 months)									
1	Observational studies (non-RCTs)	Serious^a^	Not serious^g^	Serious^h^	Serious^g^	27		⨁◯◯◯ Very low	This study demonstrated a statistically significant improvements, effect size: 0.3; CI 10.143–16.857; (*p* < 0.000)
2	Observational studies (non-RCTs)	Serious^a^	Not serious^g^	Serious^h^	Serious^g^	376		⨁◯◯◯ Very low	Both studies reported statistically significant improvements, effect size for Ru et al. (effect size: 0.2 CI 180.202–184.789; *p* = 0.001) and Chinchai et al. 2021 (effect size: 0.4, CI − 7.643–18.643; *p* = 0.02)
Quality of life (assessed with WHOQOL–BREF; follow-up: 3 months)									
2	Observational studies (non-RCTs)	Very serious^i^	Not serious^d^	Serious^j^	Serious^d^	55		⨁◯◯◯ Very low	One study reported statistically significant improvement, Chinchai et al. 2020 (effect size of 1.3; CI 8.492–16.508 ([*p* < 0.05])

Grading: no serious concerns exist, do not downgrade quality from baseline quality (e.g., for RCTs); serious concern exists, downgrade the evidence one level, e.g., from high to moderate (− 1); very serious concern exists, downgrade the evidence two levels, e.g., from high to low (− 2)

Quality of the evidence: ⊕⊕⊕⊕ High: we are very confident that the true effect lies close to that of the estimate of the effect; ⊕⊕⊕◯ Moderate: we are moderately confident in the effect estimate: the true effect is likely to be close to the estimate of the effect, but there is a possibility that it is substantially different; ⊕⊕◯◯ Low: our confidence in the effect estimate is limited: the true effect may be substantially different from the estimate of the effect; ⊕◯◯◯ Very low: we have very little confidence in the effect estimate: the true effect is likely to be substantially different from the estimate of effect

*FAC* functional ambulation category, *WHODAS* World Health Organization disability assessment schedule, *CI* confidence interval, *BI* Barthel Index, *WHOQOL* World Health Organization Quality of Life, *BADL* basic activities of daily living, *WHOQOL–BREF* World health organization quality of life–BREF

^a^Most information is from studies at a low risk of bias (blinded outcome assessors)

^b^Studies used various tools to measure the outcome, and only one study demonstrated an effect

^c^Assessed different populations, same interventions and comparison (usual care), and outcome

^d^Studies used the same tools to measure the outcome, and only one study demonstrated an effect

^e^Assessed same populations, same interventions and comparison (usual care) and outcome

^f^Studies used various tools, and none of the studies demonstrated an effect

^g^Studies used various tools to measure the outcome, and both demonstrated an effect

^h^Assessed the same populations, interventions, and outcomes (ADLs)

^i^Most information is from studies at low or unclear risk of bias

^j^Assessed different populations, same interventions and outcome

### Effects on outcomes

In this section, we first detail the effects of mobility and ADL outcomes (our primary study question) and those related to our secondary study questions.

#### Effects on physical functioning (mobility, ADLs): interventions by family caregivers

Two RCTs [24, 26], totaling 1494 stroke participants, assessed the impact of family intervention to improve mobility (Table 1a). One study [24] demonstrated a statistically significant improvement in mobility for those randomized to intervention after adjusting for confounders, with a small effect size of 0.3; confidence interval (CI) 121.81–122.19; (*p* = 0.04) (Table 1a). In contrast, the other study found no statistically significant difference in mobility outcomes [26].

Three RCTs [24–26], totaling 1555 stroke participants, reported on interventions by family caregivers to improve ADL outcomes. These studies used various tools to measure ADLs (Barthel Index and the Nottingham extended ADL scale) and one of the three studies that used the Barthel Index [25] demonstrated a statistically significant improvement in ADL for the intervention group (unadjusted analysis), with a small effect size of 0.4; CI 25.92–35.08; (*p* = 0.03) (Table 1a).

#### Effects on physical functioning (mobility, ADLs): interventions by community health workers or volunteers

One RCT [27] with 76 participants with HIV/AIDs assessed interventions by CHWs and found no statistically significant difference in mobility using various outcomes [27].

Of the three non-RCTs, one study with interventions by village health volunteers (VHVs) demonstrated a statistically significant improvement in mobility at post-test versus pre-test, with an effect size of 0.3; 10.143–16.857; (*p* < 0.05) among stroke patients [32].

Of the two non-RCTs, one with 365 participants that investigated interventions to improve ADL outcomes by community rehabilitation workers [29] demonstrated a statistically significant improvement for the intervention group compared to the control (effect size 0.2; CI 180.202–184.789; [*p* < 0.001]). Another pre-and post-study by VHVs [30] among eleven stroke participants demonstrated a statistically significant improvement after the intervention was compared to baseline (effect size of 0.4; CI − 7.643–18.643; [*p* = 0.026]) (Table 1b) [30].

#### Effects on quality of life: interventions by family caregivers

Two RCTs, totaling 305 participants, investigated interventions by family caregivers and reported no greater effect of the intervention on QOL outcomes (using the EuroQol-5D) (Table 1a) [24, 25].

#### Effects on quality of life: interventions by community health workers or volunteers

One RCT with interventions by CHWs, totaling 76 participants, reported no effect of the intervention on improving QOL outcomes [27] (Table 1a).

Of the two non-RCTs that reported on QOL outcomes, a pre–post study by VHVs demonstrated a statistically significant improvement with a large effect size of 1.3; CI 8.492–16.508; (*p* < 0.000) [31]. In contrast, an observational comparison of two interventions (community compared with the hospital) did not demonstrate a difference [33] (Table 1b).

No evidence was found for other outcomes, such as social participation or changes in processes of interest.

#### Characteristics of the interventions by family members/caregivers that demonstrated an effect

Mobility outcomes were improved (adjusted analysis) in one RCT of an intervention by family caregivers once trained in-hospital by nurses for 3 days, 15–30 min, followed by phone calls every 2–4 weeks after hospital discharge [24]; caregivers were recommended to support patients regularly for 8 weeks.

ADL outcomes were improved in an RCT study, where nurses provided the caregivers’ training in-hospital for 60 min once a day, three times, followed by a teach-back technique to assess if the caregivers had mastered the training [25].

#### Characteristics of the interventions by community health workers or volunteers that demonstrated an effect

Mobility outcomes were improved in an observational study by VHVs, trained at the community rehabilitation centers by rehabilitation professionals for 7 h in 1 day (3 h of theory and 4 h of practical sessions) [32]. VHVs were given a manual with pictures and explanations that were easy to read (e.g., by those not in the medical field) and were required to conduct home visits once weekly (1 h per visit) for 8 consecutive weeks.

For the non-RCTs that reported on ADL outcomes, the intervention was delivered by rehabilitation professionals trained community rehabilitation workers in groups in community rehabilitation centers [29], while VHVs provided the intervention in patients’ homes in the other [30]. The interventions in both non-RCT studies were provided twice a week for 1–1.5 h, with at-home practice expected five times per week for 1.5 h [29]. The programs lasted eight [30] to 12 weeks [29]. ADL outcomes in both studies were improved.

Finally, for QoL outcomes, the non-RCT study that demonstrated improvements in this measure type [31] used interventions delivered by VHVs, who were trained by rehabilitation professionals for 10 h in 1 day (4 h theory and 6 h practical sessions). A manual detailing the intervention with pictures and explanations was provided to VHVs and families of stroke patients; VHVs were required to score 80% or more on their intervention skills to provide rehabilitation services. Patients were expected to participate in the rehabilitation program in the community rehabilitation center twice a week, 1.5 h each time, for 3 months [31].

No study among those reporting improvements in mobility or ADL outcomes provided details about the expected time or amount (i.e., dose) of rehabilitation activities conducted with or by the patient.

## Discussion

This review synthesizes the evidence of the effectiveness of health-related outcomes of basic physical rehabilitation interventions delivered to adults with physical impairments by non-professional community-level workers or informal caregivers using a task-shifting or task-sharing approach in the community. Ten studies were included, of which five were RCTs. Studies were mainly conducted in Asia (*n* = 6), most commonly with stroke survivors (*n* = 8), family caregivers were most frequently used to deliver the intervention (*n* = 4), and the intervention was usually provided in the patient’s homes (*n* = 7), with training initiated in the hospital by health professionals (*n* = 4). A total of 2149 participants were involved in these studies.

The results of the studies included in this review were inconclusive, either due to the mixed findings (e.g., small effect sizes to no effect) or the methodological shortcomings (e.g., graded evidence all appraised as *low* to *very low* confidence, even when arising from RCTs).

Compared with usual care, non-professional community-level workers and informal caregivers delivered physical rehabilitation interventions did not consistently improve mobility, ADLs, or QOL. Interestingly none of the studies that demonstrated benefits in either mobility, ADLs, or QOL had an effect in any other domain. Although there is inconsistency in outcomes and methodological weaknesses, reported characteristics of interventions that demonstrated to have an effect were those with the expertise of the trainers (i.e., skilled professionals), the amount of training for intervention providers, and a prescribed home practice plan.

Although Zhou et al. [24] demonstrated some effect of the intervention on mobility outcomes, the authors noted that the way nurses were tasked to train family caregivers on rehabilitation interventions was not optimal, as nurses were just asked to accumulate a new set and tasks and skills into their loaded schedules. Lack of rehabilitation intervention expertise may have also accounted for the lack of effect on other domains, including ADLs. In addition to the trainer’s expertise, it is important to consider the amount of training for intervention providers. Two studies [24, 26], commented that the amount of training provided was inadequate. On average, these studies provided 45 min for three training days for intervention providers (i.e., enabling task-shifting or task-sharing).

When training those without experience with rehabilitation interventions, it is important to allow enough time and practice for intervention providers to become comfortable with the intervention, and to test for fidelity of the intervention delivery. Keeping interventions simple and providing follow-up training opportunities are also important means to improve intervention fidelity [24, 26]. Although few studies described the amount of practice intended with the patient (i.e., the dose of intervention), a prescribed home practice plan, coupled with regular follow-up, may contribute to the ability first to assess and then improve the intervention fidelity. Overall, fidelity issues need to be addressed to ascertain better the effectiveness of task-shifting and task-sharing interventions for the delivery of basic rehabilitation in the community.

In addition to those lessons learned from the studies included in this review, there are other novel approaches to intervention design and delivery that may also improve the effectiveness of these interventions. For example, digital health technologies, especially those that are low-cost and easy to use, might facilitate training [14, 15]. A recent study of the use of an mHealth strategy by CBR workers in India compared to control, showed that the CBR workers who used the mHealth strategy were more confident and able to implement adaptive feeding interventions for families of children with cerebral palsy better than their counterparts in the control group [34]. Moreover, this approach was preceded by a culturally sensitive needs assessment that was used to inform the training modules [35] and mHealth support given to the CBR workers in the active group. Such an approach aligns with the recognized need to account for the socio-cultural milieu and overall cultural acceptability of the approaches that may enable community-level workers to deliver task-sharing strategies more effectively. In addition, digital health technologies could help to improve supervision and the amount of at-home practice [36, 37].

We may also be able to improve the provision of the intervention as well as the amount of practice using primary care services to initiate, refer to, and provide basic rehabilitation services in low-resource settings [38, 39]. While the evidence-base for doing so is still on its infancy, research, and development on improving integration of rehabilitation services into primary care (with the subsequent improved outreach to local populations) is an agenda that the WHO has been pushing forward as one that is likely feasible and efficient to make basic rehabilitation available to underserved populations [38, 39]. Aligned with that call, a recent research report from South Africa unraveled a 10-year process that led to rehabilitation referral recommendations being considered for inclusion in South Africa’s primary health care guidelines which, albeit with hurdles, indeed increased referrals to rehabilitation from primary health care [40].

In summary, carefully considering by whom and how non-professional community-level workers or informal caregivers are trained, keeping interventions simple, and clearly defining the type and amount of practice are important considerations and may be key in determining whether task-sharing approaches are effective. In addition, digital technology, context-sensitive training materials, and rehabilitation-inclusive primary care structures are also potential considerations to improve the quality of rehabilitation interventions delivered through task-sharing.

This review demonstrated that research into the effectiveness of non-professional community-level workers and informal caregivers providing rehabilitation interventions is starting, and suboptimal methodological quality may contribute to a lack of consistency in results. It is key to ensure that more robust studies are designed and implemented to enhance the body of knowledge in this area [41]. In addition, this review identified that Asian countries and stroke patients were the most frequently studied geographical contexts and patient populations. Even though we were open to include and indeed locate papers addressing low-resource settings of high-income countries, we found none fully met our eligibility criteria. Therefore, there is likely a need to enlarge the contexts, in countries across income levels, under which task-sharing approaches for delivering community-level rehabilitation interventions are being studied to meet the rehabilitation needs of underserved populations.

## Limitations

The review had a variety of limitations. First, titles and abstracts needed to be in English, French, Spanish, or Portuguese, and the searches were conducted in English, which may lead to a suboptimal representation of studies reported in other languages. To partly offset this limitation, as well as the insufficiencies of scientific database searches, we approached three relevant external scholars as key informants—with expertise across three resource-poor world regions—for identifying any additional studies, including those of local scope. Second, we could not extract data from the studies that indicated the details about the expected amount of practice and progression of skills by the patient, which might affect the replicability of these interventions as well as their comparison in this systematic review. Finally, meta-analyses or sub-group analyses were not possible due to the heterogeneity of the studies (in study design, outcome measures, intervention details, and implementation strategies).

## Conclusion

While task-sharing is a possible strategy to increase access to unmet basic rehabilitation needs in low-resource settings, the current evidence on the effectiveness of delivery of rehabilitation interventions by non-professional community-level workers and informal caregivers is inconclusive. We can use the data and experiences from existing studies to better design studies and improve the implementation of interventions. We can also consider novel approaches to improve training and adherence to the intervention. While the results of this review show that the data are inconsistent, there are important lessons from positive as well as neutral studies to improve both study and intervention design in future studies.

## Supplementary Information

**Additional file 1: Appendix S1.** PRISMA checklist. **Appendix S2.** Detailed search strategy for all databases. **Appendix S3.** Risk of bias for RCTs. **Appendix S4.** Risk of bias for non-RCTs.

## Data Availability

The data sets used and or analyzed during the current study are available from the corresponding author (AK) on reasonable request.

## Abbreviations

LMICs: Low and Middle Income Countries
CHWs: Community health workers
VHVs: Village health volunteers
CBR: Community-based rehabilitation
ADLs: Activities of daily living
QOL: Quality of life
ROBINS-1: Risk of bias in non-randomised studies of interventions
GRADE: Grading of Recommendations Assessment, Development and Evaluation
RCTs: Randomized Control Trials
