# Supplementary material for: Effectiveness of interventions by non-professional community-level workers or family caregivers to improve outcomes for physical impairments or disabilities in low resource settings: systematic review of task-sharing strategies
Source: Hum Resour Health. 2023 Jun 21;21:48. doi: 10.1186/s12960-023-00831-7 (PMC10286375; doi:10.1186/s12960-023-00831-7)
Supplement: Supplementary file 1 — Additional file 1: Appendix S1. PRISMA checklist. Appendix S2. Detailed search strategy for all databases. Appendix S3. Risk of bias for RCTs. Appendix S4. Risk of bias for non-RCTs. [file 12960_2023_831_MOESM1_ESM.docx]

Appendices

Appendix S1: PRISMA checklist

| **Section and Topic** | **Item #** | **Checklist item** | **Location where item is reported** |
| --- | --- | --- | --- |
| **TITLE** | | |  |
| Title | 1 | Identify the report as a systematic review. | 1 |
| **ABSTRACT** | | |  |
| Abstract | 2 | See the PRISMA 2020 for Abstracts checklist. | 1 |
| **INTRODUCTION** | | |  |
| Rationale | 3 | Describe the rationale for the review in the context of existing knowledge. | 2 |
| Objectives | 4 | Provide an explicit statement of the objective(s) or question(s) the review addresses. | 3 |
| **METHODS** | | |  |
| Eligibility criteria | 5 | Specify the inclusion and exclusion criteria for the review and how studies were grouped for the syntheses. | 4 |
| Information sources | 6 | Specify all databases, registers, websites, organisations, reference lists and other sources searched or consulted to identify studies. Specify the date when each source was last searched or consulted. | 3 |
| Search strategy | 7 | Present the full search strategies for all databases, registers and websites, including any filters and limits used. | 3 |
| Selection process | 8 | Specify the methods used to decide whether a study met the inclusion criteria of the review, including how many reviewers screened each record and each report retrieved, whether they worked independently, and if applicable, details of automation tools used in the process. | 4-5 |
| Data collection process | 9 | Specify the methods used to collect data from reports, including how many reviewers collected data from each report, whether they worked independently, any processes for obtaining or confirming data from study investigators, and if applicable, details of automation tools used in the process. | 5 |
| Data items | 10a | List and define all outcomes for which data were sought. Specify whether all results that were compatible with each outcome domain in each study were sought (e.g. for all measures, time points, analyses), and if not, the methods used to decide which results to collect. | 4 |
|  | 10b | List and define all other variables for which data were sought (e.g. participant and intervention characteristics, funding sources). Describe any assumptions made about any missing or unclear information. | 4 |
| Study risk of bias assessment | 11 | Specify the methods used to assess risk of bias in the included studies, including details of the tool(s) used, how many reviewers assessed each study and whether they worked independently, and if applicable, details of automation tools used in the process. | 5 |
| Effect measures | 12 | Specify for each outcome the effect measure(s) (e.g. risk ratio, mean difference) used in the synthesis or presentation of results. | NA |
| Synthesis methods | 13a | Describe the processes used to decide which studies were eligible for each synthesis (e.g. tabulating the study intervention characteristics and comparing against the planned groups for each synthesis (item #5)). | 5 |
|  | 13b | Describe any methods required to prepare the data for presentation or synthesis, such as handling of missing summary statistics, or data conversions. | 5 |
|  | 13c | Describe any methods used to tabulate or visually display results of individual studies and syntheses. | 6 |
|  | 13d | Describe any methods used to synthesize results and provide a rationale for the choice(s). If meta-analysis was performed, describe the model(s), method(s) to identify the presence and extent of statistical heterogeneity, and software package(s) used. | NA |
|  | 13e | Describe any methods used to explore possible causes of heterogeneity among study results (e.g. subgroup analysis, meta-regression). | NA |
|  | 13f | Describe any sensitivity analyses conducted to assess robustness of the synthesized results. | NA |
| Reporting bias assessment | 14 | Describe any methods used to assess risk of bias due to missing results in a synthesis (arising from reporting biases). |  |
| Certainty assessment | 15 | Describe any methods used to assess certainty (or confidence) in the body of evidence for an outcome. | 5 |
| **RESULTS** | | |  |
| Study selection | 16a | Describe the results of the search and selection process, from the number of records identified in the search to the number of studies included in the review, ideally using a flow diagram. | 6 |
|  | 16b | Cite studies that might appear to meet the inclusion criteria, but which were excluded, and explain why they were excluded. |  |
| Study characteristics | 17 | Cite each included study and present its characteristics. | 6 |
| Risk of bias in studies | 18 | Present assessments of risk of bias for each included study. | 6 |
| Results of individual studies | 19 | For all outcomes, present, for each study: (a) summary statistics for each group (where appropriate) and (b) an effect estimate and its precision (e.g. confidence/credible interval), ideally using structured tables or plots. | 6-7 |
| Results of syntheses | 20a | For each synthesis, briefly summarise the characteristics and risk of bias among contributing studies. | 6 |
|  | 20b | Present results of all statistical syntheses conducted. If meta-analysis was done, present for each the summary estimate and its precision (e.g. confidence/credible interval) and measures of statistical heterogeneity. If comparing groups, describe the direction of the effect. | NA |
|  | 20c | Present results of all investigations of possible causes of heterogeneity among study results. | NA |
|  | 20d | Present results of all sensitivity analyses conducted to assess the robustness of the synthesized results. | NA |
| Reporting biases | 21 | Present assessments of risk of bias due to missing results (arising from reporting biases) for each synthesis assessed. | NA |
| Certainty of evidence | 22 | Present assessments of certainty (or confidence) in the body of evidence for each outcome assessed. | 6 |
| **DISCUSSION** | | |  |
| Discussion | 23a | Provide a general interpretation of the results in the context of other evidence. | 8-9 |
|  | 23b | Discuss any limitations of the evidence included in the review. | 10 |
|  | 23c | Discuss any limitations of the review processes used. | 10 |
|  | 23d | Discuss implications of the results for practice, policy, and future research. | 9-10 |
| **OTHER INFORMATION** | | |  |
| Registration and protocol | 24a | Provide registration information for the review, including register name and registration number, or state that the review was not registered. | 3 |
|  | 24b | Indicate where the review protocol can be accessed, or state that a protocol was not prepared. | 3 |
|  | 24c | Describe and explain any amendments to information provided at registration or in the protocol. | 4 |
| Support | 25 | Describe sources of financial or non-financial support for the review, and the role of the funders or sponsors in the review. | 11 |
| Competing interests | 26 | Declare any competing interests of review authors. | 11 |
| Availability of data, code and other materials | 27 | Report which of the following are publicly available and where they can be found: template data collection forms; data extracted from included studies; data used for all analyses; analytic code; any other materials used in the review. |  |

Appendix S2: search strategy

Search Report – All Scientific Databases

February 18th, 2022

Total entries: 1185 with duplicates; 1011 with the EndNote automatic deduplication

**PubMed: (210 entries)**

("Adult"[Mesh] NOT "Child"[Mesh] NOT "Infant"[Mesh]) **AND** ("Community Health Workers"[Mesh] OR "community health worker"[Text Word] OR "community based rehabilitation"[Text Word] OR "community intervention*"[Text Word] OR "Home Health Aides"[Mesh] OR "community health agent"[Text Word] OR "lay health worker"[Text Word] OR "Caregivers"[Mesh] OR "Family-led"[Text Word] OR "Community Support"[Mesh] OR "community health assistant*"[Text Word] OR "cadre"[Text Word] OR "volunteer health worker"[Text Word] OR "Homemaker Services"[Mesh] OR "Delegation, Professional"[Mesh] OR "Task shifting"[Text Word] OR "Task sharing"[Text Word]) **AND** ("Rehabilitation"[Subheading] OR "Rehabilitation"[Mesh] OR "Rehabilitation"[tw] OR "Recovery of Function"[Mesh] OR "Activities of Daily Living"[Mesh] OR "Self Care"[Mesh] OR "continuity of care"[Text Word] OR "community integration"[Text Word] OR "community support"[Text Word] OR "Disability Evaluation"[Mesh] OR "Disabled Persons"[Mesh] OR "Disability Studies"[Mesh] OR "disability"[Text Word] OR "disabilities"[Text Word] OR "impairment"[Text Word] OR "impairments"[Text Word] OR "Self-Management"[Mesh]) **AND** ("Developing Countries"[Mesh] OR "low and middle income"[Text Word] OR "Argentina"[Mesh] OR "Africa"[Mesh] OR ("China"[Mesh] NOT "Hong Kong"[Mesh] NOT "Macau"[Mesh]) OR "Democratic People's Republic of Korea"[Mesh] OR "Mongolia"[Mesh] OR "Asia, Central"[Mesh] OR "Asia, Northern"[Mesh] OR "Bangladesh"[Mesh] OR "Bhutan"[Mesh] OR "India"[Mesh] OR "Nepal"[Mesh] OR "Pakistan"[Mesh] OR "Sri Lanka"[Mesh] OR "Afghanistan"[Mesh] OR "Iran"[Mesh] OR "Iraq"[Mesh] OR "Jordan"[Mesh] OR "Lebanon"[Mesh] OR "Syria"[Mesh] OR "Turkey"[Mesh] OR "Yemen"[Mesh] OR "Cambodia"[Mesh] OR "Indonesia"[Mesh] OR "Laos"[Mesh] OR "Malaysia"[Mesh] OR "Myanmar"[Mesh] OR "Philippines"[Mesh] OR "Thailand"[Mesh] OR "Timor-Leste"[Mesh] OR "Vietnam"[Mesh] OR "Mexico"[Mesh] OR "Belize"[Mesh] OR "Costa Rica"[Mesh] OR "El Salvador"[Mesh] OR "Guatemala"[Mesh] OR "Honduras"[Mesh] OR "Nicaragua"[Mesh] OR "Cuba"[Mesh] OR "Dominica"[Mesh] OR "Dominican Republic"[Mesh] OR "Grenada"[Mesh] OR "Haiti"[Mesh] OR "Jamaica"[Mesh] OR "Saint Lucia"[Mesh] OR "Saint Vincent and the Grenadines"[Mesh] OR "Bolivia"[Mesh] OR "Brazil"[Mesh] OR "Colombia"[Mesh] OR "Ecuador"[Mesh] OR "Paraguay"[Mesh] OR "Peru"[Mesh] OR "Suriname"[Mesh] OR "Venezuela"[Mesh] OR "Albania"[Mesh] OR "Bosnia and Herzegovina"[Mesh] OR "Bulgaria"[Mesh] OR "Kosovo"[Mesh] OR "Republic of North Macedonia"[Mesh] OR "Montenegro"[Mesh] OR "Moldova"[Mesh] OR "Republic of Belarus"[Mesh] OR "Serbia"[Mesh] OR "Ukraine"[Mesh] OR "Transcaucasia"[Mesh] OR "Kazakhstan"[Mesh] OR "Kyrgyzstan"[Mesh] OR "Uzbekistan"[Mesh] OR "Samoa"[Mesh] OR ("Micronesia"[Mesh] NOT "Guam"[Mesh] NOT "Palau"[Mesh]) OR "Fiji"[Mesh] OR "Papua New Guinea"[Mesh] OR "Vanuatu"[Mesh] OR "Tonga"[Mesh]) **AND** ("Study Characteristics" [Publication Type] OR "Feasibility Studies"[Mesh] OR "Pilot Projects"[Mesh] OR "Support of Research" [Publication Type]) **AND** ("intervention"[Text Word] OR "program"[Text Word] OR "trial"[Text Word] or "delivery"[Text Word] OR "training"[Text Word] OR "education"[Text Word] OR "Rehabilitation"[Subheading] OR "Rehabilitation"[Mesh]) **NOT** ("Mentally Ill Persons"[MeSH] OR "Psychiatry"[Mesh] OR "Psychiatric Nursing"[Mesh] OR “Mental Disorders”[Mesh] OR “Mental Health Recovery”[Mesh] NOT “Psychiatric Rehabilitation”[Mesh])

**CENTRAL (172 entries)**

**Filtered for:** Trials

("Community Health Workers" OR "community based rehabilitation” OR "lay health worker" OR "Caregiver-led" OR "Family-led" OR "Caregiver-delivered" OR "Family-delivered" OR "community health assistants" OR "cadre" OR "volunteer worker" OR "Task shifting" OR "Task sharing") **AND** ("Rehabilitation" OR Disability OR Disabilities OR "impairment" OR "impairments") **AND** ( intervention OR program OR trial OR delivery OR training OR education) NOT (mental OR psychiatric OR child OR children OR schizophrenia OR depression OR diabetes) **AND** (“low income country” OR “low income countries” OR “middle income country” OR “middle income countries” OR “developing country” OR “developing countries” OR Africa OR Angola OR Algeria OR Bangladesh OR Belize OR Benin OR Bhutan OR Bolivia OR Cabo Verde OR Cambodia OR Cameroon OR Comoros OR Congo, Rep. OR Côte d'Ivoire OR Djibouti OR Egypt, Arab Rep. OR El Salvador OR Eswatini OR Ghana OR Haiti OR Honduras OR India OR Indonesia OR Iran, Islamic Rep OR Kenya OR Kiribati OR Kyrgyz Republic OR Lao PDR OR Lesotho OR Mauritania OR Micronesia, OR Mongolia OR Morocco OR Myanmar OR Nepal OR Nicaragua OR Nigeria OR Pakistan OR Papua New Guinea OR Philippines OR Samoa OR São Tomé and Principe OR Senegal OR Solomon Islands OR Sri Lanka OR Tanzania OR Tajikistan OR Timor-Leste OR Tunisia OR Ukraine OR Uzbekistan OR Vanuatu OR Vietnam OR West Bank and Gaza OR Zambia OR Zimbabwe OR Albania OR American Samoa OR Argentina OR Armenia OR Azerbaijan OR Belarus OR Bosnia and Herzegovina OR Botswana OR Brazil OR Bulgaria OR China OR Colombia OR Costa Rica OR Cuba OR Dominica OR Dominican Republic OR Equatorial Guinea OR Ecuador OR Fiji OR Gabon OR Georgia OR Grenada OR Guatemala OR Guyana OR Iraq OR Jamaica OR Jordan OR Kazakhstan OR Kosovo OR Lebanon OR Libya OR Malaysia OR Maldives OR Marshall Islands OR Mauritius OR Mexico OR Moldova OR Montenegro OR Namibia OR North Macedonia OR Panama OR Paraguay OR Peru OR Romania OR Russian Federation OR Serbia OR South Africa OR St. Lucia OR St. Vincent and the Grenadines OR Suriname OR Thailand OR Tonga OR Turkey OR Turkmenistan OR Tuvalu OR Afghanistan OR Burkina Faso OR Burundi OR Central African Republic OR Chad OR Congo, Rep. OR Eritrea OR Ethiopia OR Gambia, The OR Guinea OR Guinea-Bissau OR Korea OR Liberia OR Madagascar OR Malawi OR Mali OR Mozambique OR Niger OR Rwanda OR Sierra Leone OR Somalia OR South Sudan OR Sudan OR Syrian Arab Republic OR Togo OR Uganda OR Yemen)

**Scopus: (176 entries )**

( TITLE-ABS-KEY ( "Community Health Worker*" ) OR TITLE-ABS-KEY ( "community based rehabilitation" ) OR ( community PRE/2 aid* ) OR ( lay PRE/2 worker* ) OR ( community PRE/2 assistant* ) OR ( volunteer PRE/2 worker* ) OR TITLE-ABS-KEY ( cadre ) OR TITLE-ABS-KEY ( "task shift*" ) OR TITLE-ABS-KEY ( "task shar*" ) OR ( family W/5 rehabilitation ) OR TITLE-ABS-KEY ( caregiver W/5 rehabilitation ) ) AND ( TITLE-ABS-KEY ( rehabilitation ) OR TITLE-ABS-KEY ( "self care" ) OR TITLE-ABS-KEY ( "Activities of Daily Living" ) OR TITLE-ABS-KEY ( "disabilit*" ) OR TITLE-ABS-KEY ( impairment ) OR TITLE-ABS-KEY ( assistive ) OR TITLE-ABS-KEY ( "self management" ) OR INDEXTERMS ( rehabilitation ) ) AND ( LIMIT-TO ( AFFILCOUNTRY , "India" ) OR LIMIT-TO ( AFFILCOUNTRY , "China" ) OR LIMIT-TO ( AFFILCOUNTRY , "South Africa" ) OR LIMIT-TO ( AFFILCOUNTRY , "Brazil" ) OR LIMIT-TO ( AFFILCOUNTRY , "Turkey" ) OR LIMIT-TO ( AFFILCOUNTRY , "Malaysia" ) OR LIMIT-TO ( AFFILCOUNTRY , "Pakistan" ) OR LIMIT-TO ( AFFILCOUNTRY , "Nigeria" ) OR LIMIT-TO ( AFFILCOUNTRY , "Uganda" ) OR LIMIT-TO ( AFFILCOUNTRY , "Russian Federation" ) OR LIMIT-TO ( AFFILCOUNTRY , "Thailand" ) OR LIMIT-TO ( AFFILCOUNTRY , "Kenya" ) OR LIMIT-TO ( AFFILCOUNTRY , "Jordan" ) OR LIMIT-TO ( AFFILCOUNTRY , "Jamaica" ) OR LIMIT-TO ( AFFILCOUNTRY , "Rwanda" ) OR LIMIT-TO ( AFFILCOUNTRY , "Papua New Guinea" ) OR LIMIT-TO ( AFFILCOUNTRY , "Viet Nam" ) OR LIMIT-TO ( AFFILCOUNTRY , "Bulgaria" ) OR LIMIT-TO ( AFFILCOUNTRY , "Cambodia" ) OR LIMIT-TO ( AFFILCOUNTRY , "Peru" ) OR LIMIT-TO ( AFFILCOUNTRY , "Benin" ) OR LIMIT-TO ( AFFILCOUNTRY , "Mozambique" ) OR LIMIT-TO ( AFFILCOUNTRY , "Togo" ) OR LIMIT-TO ( AFFILCOUNTRY , "Tunisia" ) OR LIMIT-TO ( AFFILCOUNTRY , "American Samoa" ) OR LIMIT-TO ( AFFILCOUNTRY , "Bolivia" ) OR LIMIT-TO ( AFFILCOUNTRY , "Ecuador" ) OR LIMIT-TO ( AFFILCOUNTRY , "Lebanon" ) OR LIMIT-TO ( AFFILCOUNTRY , "Senegal" ) OR LIMIT-TO ( AFFILCOUNTRY , "Albania" ) OR LIMIT-TO ( AFFILCOUNTRY , "Algeria" ) OR LIMIT-TO ( AFFILCOUNTRY , "Barbados" ) OR LIMIT-TO ( AFFILCOUNTRY , "Gabon" ) OR LIMIT-TO ( AFFILCOUNTRY , "Germany (Democratic Republic, DDR)" ) OR LIMIT-TO ( AFFILCOUNTRY , "Kazakhstan" ) OR LIMIT-TO ( AFFILCOUNTRY , "Mauritania" ) OR LIMIT-TO ( AFFILCOUNTRY , "Myanmar" ) OR LIMIT-TO ( AFFILCOUNTRY , "Nicaragua" ) OR LIMIT-TO ( AFFILCOUNTRY , "Niger" ) OR LIMIT-TO ( AFFILCOUNTRY , "Russia" ) OR LIMIT-TO ( AFFILCOUNTRY , "Seychelles" ) OR LIMIT-TO ( AFFILCOUNTRY , "Solomon Islands" ) OR LIMIT-TO ( AFFILCOUNTRY , "Somalia" ) OR LIMIT-TO ( AFFILCOUNTRY , "Suriname" ) OR LIMIT-TO ( AFFILCOUNTRY , "Trinidad and Tobago" ) OR LIMIT-TO ( AFFILCOUNTRY , "Uzbekistan" ) ) AND ( LIMIT-TO ( DOCTYPE , "ar" ) ) AND ( LIMIT-TO ( SUBJAREA , "HEAL" ) OR LIMIT-TO ( SUBJAREA , "PSYC" ) OR LIMIT-TO ( SUBJAREA , "NURS" ) OR EXCLUDE ( SUBJAREA , "ENGI" ) OR EXCLUDE ( SUBJAREA , "AGRI" ) OR EXCLUDE ( SUBJAREA , "BIOC" ) OR EXCLUDE ( SUBJAREA , "DENT" ) OR EXCLUDE ( SUBJAREA , "EART" ) ) AND ( EXCLUDE ( EXACTKEYWORD , "Child" ) OR EXCLUDE ( EXACTKEYWORD , "Depression" ) OR EXCLUDE ( EXACTKEYWORD , "Schizophrenia" ) OR EXCLUDE ( EXACTKEYWORD , "Adolescent" ) OR EXCLUDE ( EXACTKEYWORD , "Mental Health" ) OR EXCLUDE ( EXACTKEYWORD , "Mental Disease" ) OR EXCLUDE ( EXACTKEYWORD , "Mental Health Service" ) ) AND ( EXCLUDE ( EXACTKEYWORD , "Qualitative Research" ) OR EXCLUDE ( EXACTKEYWORD , "Cross-sectional Study" ) ) **AND** ( TITLE-ABS-KEY (intervention) OR TITLE-ABS-KEY (program) OR TITLE-ABS-KEY (trial) OR TITLE-ABS-KEY (delivery) OR TITLE-ABS-KEY (training) OR TITLE-ABS-KEY (education) OR TITLE-ABS-KEY (Rehabilitation))

**Global Health - EBSCO (61 entries)**

**Expanders**

- [**X**](https://web-p-ebscohost-com.turing.library.northwestern.edu/ehost/breadbox/remove?item=expander_thesaurus&sid=825e5e82-4d45-48da-a575-2d78c7c5fde6%40redis&vid=7)Apply related words
- [**X**](https://web-p-ebscohost-com.turing.library.northwestern.edu/ehost/breadbox/remove?item=expander_enhancedsubjectprecision&sid=825e5e82-4d45-48da-a575-2d78c7c5fde6%40redis&vid=7)Apply equivalent subjects

**Limiters**

- [**X**](https://web-p-ebscohost-com.turing.library.northwestern.edu/ehost/breadbox/remove?item=limiter_RV&sid=825e5e82-4d45-48da-a575-2d78c7c5fde6%40redis&vid=7)Peer Reviewed

("Community Health Worker*" OR "community based rehabilitation” OR "lay worker*" OR "Caregiver-led" OR "Family-led" OR "Caregiver-delivered" OR "Family-delivered" OR "community health assistant*" OR "cadre" OR "volunteer " OR "Task shifting" OR "Task sharing") **AND** ("Rehabilitation" OR “Activities of daily living” OR “Self care” OR Disability OR Disabilities OR "impairment" OR "impairments") **AND** ( intervention OR program OR trial OR delivery OR training OR education) NOT (mental OR psychiatric OR child OR children OR schizophrenia OR diabetes) **AND** (“low income country” OR “low income countries” OR “middle income country” OR “middle income countries” OR “developing country” OR “developing countries” OR Africa OR Angola OR Algeria OR Bangladesh OR Belize OR Benin OR Bhutan OR Bolivia OR Cabo Verde OR Cambodia OR Cameroon OR Comoros OR Congo, Rep. OR Côte d'Ivoire OR Djibouti OR Egypt, Arab Rep. OR El Salvador OR Eswatini OR Ghana OR Haiti OR Honduras OR India OR Indonesia OR Iran, Islamic Rep OR Kenya OR Kiribati OR Kyrgyz Republic OR Lao PDR OR Lesotho OR Mauritania OR Micronesia, OR Mongolia OR Morocco OR Myanmar OR Nepal OR Nicaragua OR Nigeria OR Pakistan OR Papua New Guinea OR Philippines OR Samoa OR São Tomé and Principe OR Senegal OR Solomon Islands OR Sri Lanka OR Tanzania OR Tajikistan OR Timor-Leste OR Tunisia OR Ukraine OR Uzbekistan OR Vanuatu OR Vietnam OR West Bank and Gaza OR Zambia OR Zimbabwe OR Albania OR American Samoa OR Argentina OR Armenia OR Azerbaijan OR Belarus OR Bosnia and Herzegovina OR Botswana OR Brazil OR Bulgaria OR China OR Colombia OR Costa Rica OR Cuba OR Dominica OR Dominican Republic OR Equatorial Guinea OR Ecuador OR Fiji OR Gabon OR Georgia OR Grenada OR Guatemala OR Guyana OR Iraq OR Jamaica OR Jordan OR Kazakhstan OR Kosovo OR Lebanon OR Libya OR Malaysia OR Maldives OR Marshall Islands OR Mauritius OR Mexico OR Moldova OR Montenegro OR Namibia OR North Macedonia OR Panama OR Paraguay OR Peru OR Romania OR Russian Federation OR Serbia OR South Africa OR St. Lucia OR St. Vincent and the Grenadines OR Suriname OR Thailand OR Tonga OR Turkey OR Turkmenistan OR Tuvalu OR Afghanistan OR Burkina Faso OR Burundi OR Central African Republic OR Chad OR Congo, Rep. OR Eritrea OR Ethiopia OR Gambia, The OR Guinea OR Guinea-Bissau OR Korea OR Liberia OR Madagascar OR Malawi OR Mali OR Mozambique OR Niger OR Rwanda OR Sierra Leone OR Somalia OR South Sudan OR Sudan OR Syrian Arab Republic OR Togo OR Uganda OR Yemen)

**Web of Science (233 entries)**

<https://www.webofscience.com/wos/woscc/summary/d1fefe40-84ff-46eb-a83a-5466a0b1ddc0-24330bb8/relevance/1>

**Or**

(TS="Community Health Worker*" OR TS="community based rehabilitation” OR TS="lay worker*" OR TS="Caregiver-led" OR TS="Family-led" OR TS="Caregiver-delivered" OR TS="Family-delivered" OR TS="community health assistant*" OR TS="cadre" OR TS="volunteer " OR TS="Task shifting" OR TS="Task sharing") AND (TS="Rehabilitation" OR TS=Disability OR TS=Disabilities OR TS="impairment" OR TS="impairments") AND (TS=intervention OR TS=program OR TS=trial OR TS=delivery OR TS=training)

*NOT Countries/Regions: USA or ENGLAND or AUSTRALIA or CANADA or NETHERLANDS or SWITZERLAND or GERMANY or SCOTLAND or ITALY or SINGAPORE or WALES or NORTH IRELAND or PORTUGAL or CZECH REPUBLIC or QATAR or CYPRUS or CROATIA or U ARAB EMIRATES or GREECE or IRELAND or SOUTH KOREA or DENMARK or NORWAY or BELGIUM or SWEDEN or SLOVENIA or LUXEMBOURG or POLAND or AUSTRIA or FINLAND or JAPAN or FRANCE or SPAIN or NEW ZEALAND or MALTA Document Types: Articles NOT Research Areas: Substance Abuse or Development Studies or Dermatology or Parasitology or Anesthesiology or Biochemistry Molecular Biology or Dentistry Oral Surgery Medicine or Mathematical Computational Biology or Nutrition Dietetics or Obstetrics Gynecology NOT Research Areas: Pediatrics NOT Web of Science Categories: Psychiatry or Education Educational Research or Education Special*

**CINAHL Plus with Full Text (207)**

#### Expanders

- [**X**](https://web-p-ebscohost-com.turing.library.northwestern.edu/ehost/breadbox/remove?item=expander_thesaurus&sid=a4ccc5b4-3928-479b-82d6-a47809e4398e%40redis&vid=15)Apply related words
- [**X**](https://web-p-ebscohost-com.turing.library.northwestern.edu/ehost/breadbox/remove?item=expander_enhancedsubjectprecision&sid=a4ccc5b4-3928-479b-82d6-a47809e4398e%40redis&vid=15)Apply equivalent subjects

#### Limiters

- [**X**](https://web-p-ebscohost-com.turing.library.northwestern.edu/ehost/breadbox/remove?item=limiter_AA1&sid=a4ccc5b4-3928-479b-82d6-a47809e4398e%40redis&vid=15)Abstract Available
- [**X**](https://web-p-ebscohost-com.turing.library.northwestern.edu/ehost/breadbox/remove?item=limiter_RV&sid=a4ccc5b4-3928-479b-82d6-a47809e4398e%40redis&vid=15)Peer Reviewed
- [**X**](https://web-p-ebscohost-com.turing.library.northwestern.edu/ehost/breadbox/remove?item=limiter_PF1&sid=a4ccc5b4-3928-479b-82d6-a47809e4398e%40redis&vid=15)Exclude Pre-CINAHL

#### Source Types

- [**X**](https://web-p-ebscohost-com.turing.library.northwestern.edu/ehost/breadbox/remove?item=sourcetype_1000AJ&sid=a4ccc5b4-3928-479b-82d6-a47809e4398e%40redis&vid=15)Academic Journals

("Community Health Worker*" OR "community based rehabilitation” OR "lay health worker*" OR "Caregiver-led" OR "Family-led" OR "Caregiver-delivered" OR "Family-delivered" OR "community health assistant*" OR "cadre" OR "volunteer " OR "Task shifting" OR "Task sharing") **AND** ("Rehabilitation" OR Disability OR Disabilities OR "impairment" OR "impairments") **AND** ( intervention OR program OR trial OR delivery OR training) NOT (mental OR psychiatric OR child OR children OR schizophrenia OR dementia OR depression OR diabetes) **AND** (“low income country” OR “low income countries” OR “middle income country” OR “middle income countries” OR “developing country” OR “developing countries” OR Africa OR Angola OR Algeria OR Bangladesh OR Belize OR Benin OR Bhutan OR Bolivia OR Cabo Verde OR Cambodia OR Cameroon OR Comoros OR Congo, Rep. OR Côte d'Ivoire OR Djibouti OR Egypt, Arab Rep. OR El Salvador OR Eswatini OR Ghana OR Haiti OR Honduras OR India OR Indonesia OR Iran, Islamic Rep OR Kenya OR Kiribati OR Kyrgyz Republic OR Lao PDR OR Lesotho OR Mauritania OR Micronesia, OR Mongolia OR Morocco OR Myanmar OR Nepal OR Nicaragua OR Nigeria OR Pakistan OR Papua New Guinea OR Philippines OR Samoa OR São Tomé and Principe OR Senegal OR Solomon Islands OR Sri Lanka OR Tanzania OR Tajikistan OR Timor-Leste OR Tunisia OR Ukraine OR Uzbekistan OR Vanuatu OR Vietnam OR West Bank and Gaza OR Zambia OR Zimbabwe OR Albania OR American Samoa OR Argentina OR Armenia OR Azerbaijan OR Belarus OR Bosnia and Herzegovina OR Botswana OR Brazil OR Bulgaria OR China OR Colombia OR Costa Rica OR Cuba OR Dominica OR Dominican Republic OR Equatorial Guinea OR Ecuador OR Fiji OR Gabon OR Georgia OR Grenada OR Guatemala OR Guyana OR Iraq OR Jamaica OR Jordan OR Kazakhstan OR Kosovo OR Lebanon OR Libya OR Malaysia OR Maldives OR Marshall Islands OR Mauritius OR Mexico OR Moldova OR Montenegro OR Namibia OR North Macedonia OR Panama OR Paraguay OR Peru OR Romania OR Russian Federation OR Serbia OR South Africa OR St. Lucia OR St. Vincent and the Grenadines OR Suriname OR Thailand OR Tonga OR Turkey OR Turkmenistan OR Tuvalu OR Afghanistan OR Burkina Faso OR Burundi OR Central African Republic OR Chad OR Congo, Rep. OR Eritrea OR Ethiopia OR Gambia, The OR Guinea OR Guinea-Bissau OR Korea OR Liberia OR Madagascar OR Malawi OR Mali OR Mozambique OR Niger OR Rwanda OR Sierra Leone OR Somalia OR South Sudan OR Sudan OR Syrian Arab Republic OR Togo OR Uganda OR Yemen)

**ProQuest (44 entries)**

mainsubject(community) AND mainsubject(rehabilitation) AND ("low income" OR "middle income") AND workers AND (intervention OR trial OR program OR training OR education) AND (Scholarly Journals OR Dissertations & Theses) NOT (mental disorders OR mental health OR children & youth OR nuclear reactions OR property disposition OR radiation hazards OR vegetation OR weapon systems OR wildlife habitat OR psychiatry OR qualitative research OR schizophrenia OR tax credits OR traffic analyses OR children OR United States--US OR Switzerland OR Canada OR Italy OR Japan OR United Kingdom--UK)

Applied Filters:

Scholarly Journals OR Dissertations & Theses

NOT (mental disorders AND mental health AND children & youth AND nuclear reactions AND property disposition AND radiation hazards AND vegetation AND weapon systems AND wildlife habitat AND psychiatry AND qualitative research AND schizophrenia AND tax credits AND traffic analyses AND children)

Article OR Dissertation/Thesis OR Case Study OR Report

NOT (United States--US AND Switzerland AND Atlanta Georgia AND California AND Canada AND Canada (Toronto) AND Italy AND Japan AND Kentucky AND New York AND Texas AND United Kingdom--UK)

**PDQ Evidence (72 entries)**

("Community Health Workers" OR "community based rehabilitation” OR "lay health worker" OR "Caregiver-led" OR "Family-led" OR "community health assistants" OR "cadre" OR "volunteer worker" OR "Task shifting" OR "Task sharing") **AND** ("Rehabilitation" OR Disability OR Disabilities OR "impairment" OR "impairments") **AND** ( intervention OR program OR trial OR delivery OR training OR education) NOT (mental OR psychiatric OR child OR children OR schizophrenia OR depression OR diabetes) **AND** (“low income country” OR “low income countries” OR “middle income country” OR “middle income countries” OR “developing country” OR “developing countries” OR Africa OR Angola OR Algeria OR Bangladesh OR Belize OR Benin OR Bhutan OR Bolivia OR Cabo Verde OR Cambodia OR Cameroon OR Comoros OR Congo, Rep. OR Côte d'Ivoire OR Djibouti OR Egypt, Arab Rep. OR El Salvador OR Eswatini OR Ghana OR Haiti OR Honduras OR India OR Indonesia OR Iran, Islamic Rep OR Kenya OR Kiribati OR Kyrgyz Republic OR Lao PDR OR Lesotho OR Mauritania OR Micronesia, OR Mongolia OR Morocco OR Myanmar OR Nepal OR Nicaragua OR Nigeria OR Pakistan OR Papua New Guinea OR Philippines OR Samoa OR São Tomé and Principe OR Senegal OR Solomon Islands OR Sri Lanka OR Tanzania OR Tajikistan OR Timor-Leste OR Tunisia OR Ukraine OR Uzbekistan OR Vanuatu OR Vietnam OR West Bank and Gaza OR Zambia OR Zimbabwe OR Albania OR American Samoa OR Argentina OR Armenia OR Azerbaijan OR Belarus OR Bosnia and Herzegovina OR Botswana OR Brazil OR Bulgaria OR China OR Colombia OR Costa Rica OR Cuba OR Dominica OR Dominican Republic OR Equatorial Guinea OR Ecuador OR Fiji OR Gabon OR Georgia OR Grenada OR Guatemala OR Guyana OR Iraq OR Jamaica OR Jordan OR Kazakhstan OR Kosovo OR Lebanon OR Libya OR Malaysia OR Maldives OR Marshall Islands OR Mauritius OR Mexico OR Moldova OR Montenegro OR Namibia OR North Macedonia OR Panama OR Paraguay OR Peru OR Romania OR Russian Federation OR Serbia OR South Africa OR St. Lucia OR St. Vincent and the Grenadines OR Suriname OR Thailand OR Tonga OR Turkey OR Turkmenistan OR Tuvalu OR Afghanistan OR Burkina Faso OR Burundi OR Central African Republic OR Chad OR Congo, Rep. OR Eritrea OR Ethiopia OR Gambia, The OR Guinea OR Guinea-Bissau OR Korea OR Liberia OR Madagascar OR Malawi OR Mali OR Mozambique OR Niger OR Rwanda OR Sierra Leone OR Somalia OR South Sudan OR Sudan OR Syrian Arab Republic OR Togo OR Uganda OR Yemen)

**Filtered for:** Primary Studies

Appendix S3: Risk of Bias for RCTs

| **Author & Year** | **Bias arising from the randomization** | **Bias due to deviations from intended intervention** | **Bias due to missing outcome data** | **Bias in measurement of the outcome** | **Bias in selection of the reported result** | **Overall bias** |
| --- | --- | --- | --- | --- | --- | --- |
| Cobbing, S. et al., 2017 | **Low risk of bias**  -stratified randomization was employed  using a computerized method to ensure an even number of male and female participants in the  intervention and control groups  - No concealment of allocations prior to assignment | **High risk of bias**  -participants and therapists were not blinded  -No deviation from the intended intervention is mentioned | **Low risk of bias**  -an intention to treat analysis was performed  -intervention group (n=38): 11% lost to follow-up (1 died, 1 moved and 2 were unable to attend)  -control group (n=38): 11% lost to follow-up (1 moved and 3 unable to contact) | **Low risk of bias**  -Outcome assessors were not aware of the intervention received by study participants | **Low risk of bias**  **-** all intended outcome measures (primary and secondary) mentioned in the protocol are completed | **Low risk of bias** |
| Lindley, R. et al., 2017 | **Low risk of bias**  -Patients were randomly assigned (1:1) to intervention or a usual care control group via a secure web-based central randomisation system with minimisation by site and stroke severity  - No information to assess concealment of allocations prior to assignment | **High risk of bias**  -participants and therapists were not blinded  -No deviation from the intended intervention is reported | **Low risk of bias**  - an intention to treat analysis was performed  -Intervention (n=623): at 6mnths: 16(3%) lost to follow-up  -Control (n=627): at 6mnths:22(4%) lost to follow-up | **Low risk of bias**  - Outcome assessors were blinded | **Low risk of bias**  - all the outcome measures mentioned in the protocol are reported in the study | **Low risk of bias** |
| Zhou, B. et al., 2019 | **Low risk of bias**  Randomized via a secure, central internet- based system, with randomly variable block sizes  No information to assess concealment of allocations prior to assignment | **High risk of bias**  -Participants and therapists were not blinded  - No deviation from the intended intervention is reported | **Low risk of bias**  - an intention to treat analysis was performed  -intervention (n=116): 2(2%) died | **Low risk of bias**  - Outcome assessors were blinded | **Low risk of bias**  - all the outcome measures mentioned in the protocol are reported | **Low risk of bias** |
| Chu, K. et al., (2020) | **Low risk of bias**  -Eligible patients were randomly  assigned either to intervention group or to control group through a secure, internet-based system  -No information to assess concealment of allocations prior to assignment | **High risk of bias**  Participants and therapists were not blinded | **High risk of bias**  An intention to treat analysis was performed  Intervention(N=31):29% (1 patient died, 4 lost to follow-up, 3 refused to participate and 1 for other reason) | **Low risk of bias**  -Outcome assessors were blinded | **Low risk of bias**  **-**All intended outcome measures (primary and secondary) mentioned in the protocol are reported in the study | **Low risk of bias** |
| Ozdemir﻿, F. et al., 2001 | **High risk of bias**  Patients were enrolled into 2 equal groups by  selecting patients consecutively, one by one, according to when  they enrolled in the study.  **-**This method of allocations patients into groups suggests lack of concealment | **High risk of bias**  Participants and therapists were not blinded | **High risk of bias**  No information to assess | **High risk of bias**  Outcome assessors were aware of the participants’ assigned intervention | **High risk of bias**  Outcomes measures are presented but no protocol to affirm if the data was collected as planned | **High risk of bias** |

Appendix S4: Risk of bias for non-RCTs

|  | ***Pre-intervention domains*** | | ***At intervention domain*** | ***Post-intervention domains*** | | | |
| --- | --- | --- | --- | --- | --- | --- | --- |
| **Author & Year** | **Bias due to confounding** | **Bias due to selection of participants** | **Bias due to classification of interventions** | **Bias due to deviations from intended interventions** | **Bias due to missing data** | **Bias in measurement of outcomes** | **Bias in selection of the reported results** |
| Ru, X. et al., 2017 | **Moderate risk** Participants in both control and intervention communities were similar and the stroke survivors  were sampled using a random-number generator | **Serious risk** Patients with severe dysfunction were unable to receive  CRAT treatment.  Accordingly, there is a possibility that the study suffered from a selection bias, which may have led to better ADLs estimate in the recruited participants and may explain the  mean baseline BI score | **No Information**  on which to base the judgment | **Serious risk**  Participants were not blinded of the intervention  -Therapists delivering the intervention were not blinded to participants’ assigned intervention  -No deviation from the intended intervention is mentioned | **Serious risk**  CRAT/intervention group (n=365): 23(6%) discontinued treatment  -No intention to treat was reported | **Serious risk**  Treatment effect evaluators were not blinded | **Serious risk**  Results from all measurements were reported but no protocol |
| Chinchai, P. et al., 2017 | **Serious risk**  -some socio-demographic data are reported    -pre and post study (we could consider pre as control) | **Serious risk**  -purposive sampling- not randomly selected  -sample:27 participants out of 4 municipalities  While invitations were sent to 10 municipalities | **No information** on which to base the judgment | **Serious risk**  -research  teams made phone calls to VHVs once every two weeks and VHV completing home visits | **No information**  on which to base the judgment.  The paper does not show any missing data (no information of patients who couldn’t complete) | **Serious risk**  - data (pre/post) were collected by different research assistants (2 OTs with 2 years’ experience in the field. | **Serious risk**  All results for the measurements were reported but no protocol to verify |
| Chinchai, P. et al., 2020 | **Serious risk**  -Some socio-demographic data are presented    - pre and post study | **Serious risk**  -purposive sampling- not randomly selected    -sample:25 out of 4 rehab centers | No information on which to base the judgment | No information on which to base the judgment | **No information** on which to base the judgment  The paper does not show any missing data (no information of patients who couldn’t complete) | **Serious risk**  -outcome data were collected by  research assistants (PTs) collecting data were unaware of the study goals | **Serious risk**  - Results from all measurements were reported as per research question but no protocol  -clinical significancy not reported? |
| Chinchai, P. et al., 2021 | **Serious risk of bias:**  - pre and post study  - no control group | **Serious risk:**  Participants were selected using purposive sampling | **No information** on which to base the judgment | **No information** on which to base the judgment | **No information** on which to base the judgment  The paper does not show any missing data (no information of patients who couldn’t complete) | Serious risk  Patients and outcome assessors were not blinded | **Serious risk:** It seems the results from all measurements were reported but no protocol |
| Balasubramanian, M.M. et al., 2012 | **Serious risk**  Substantive difference in mean age (CBR:53 vs. IBR: 37)  -Sample of only men  -No socio-demographic data presented | **Serious risk**  - purposive sampling- not randomly selected from a comprehensive list of target individuals (not representative) | **Serious risk**  -IBR vs CBR but the type of care/interventions are not specified)  -possible that patients were in both programs or recently quit IBR for CBR for example | **No information** on which to base the judgment | **No information** on which to base the judgment | **Serious risk**  -2 outcome measures (self-reported) are presented    -Likely staff knew allocation  -Period in the program not reported | **Serious risk**  -presented some of the domains of FIM and WHOQOL and presented overall summary  -no protocol to verify |
